# Second Language Interference during First Language Processing by Arabic–English Bilinguals

**DOI:** 10.3389/fpsyg.2017.01956

**Published:** 2017-11-07

**Authors:** Tahani Alsaigh, Shelia M. Kennison

**Affiliations:** Department of Psychology, Oklahoma State University, Stillwater, OK, United States

**Keywords:** bilingualism, L2 interference, Arabic–English bilinguals, bilingual memory, Saudi Arabia

## Abstract

The research investigated whether a bilinguals’ second language (L2) is activated during a task involving only the first language (L1). We tested the hypothesis that the amount of L2 interference can vary across settings, with less interference occurring in testing locations where L2 is rarely used. In Experiment 1, we compared language processing for 50 Arabic–English bilinguals tested in Saudi Arabia and 49 Arabic–English tested in the United States. In the task, participants viewed a picture and judged whether a phoneme presented over headphones was part of the L1 picture name. The results showed no effect of testing location on processing. For both groups of bilinguals, we observed L2 interference in mean error rates, but not in mean response times. We also found evidence for L2 interference in correlational analyses between response times and (a) participants’ weekly L2 usage and (b) frequency of English picture names. A second experiment with 24 Arabic monolinguals supported the conclusion that the results with bilinguals were due to L2 interference. Implications for theories of bilingual memory are discussed.

## Introduction

The nature of bilingual memory remains poorly understood, specifically with regard to whether a bilingual’s two languages are stored separately in memory or together (Grosjean, 1982, 2010; Kroll and Stewart, 1994; Dijkstra and Van Heuven, 2002; Altarriba and Isurin, 2013; Heredia and Altarriba, 2014). Some researchers advocate for separation of languages in bilingual memory (e.g., the revised hierarchical model or RHM, Kroll and Stewart, 1994). Other researchers claim that during processing, there is generally co-activation of both languages during all language processing (e.g., the bilingual interactive activation plus or BIA+ model, Dijkstra and Van Heuven, 2002). It remains unclear whether the activation of each of a bilingual’s languages varies across situational contexts. The possibility is suggested by Grosjean’s (1982, 2010) in his language mode theory. The focus of the present research was to explore the extent to which the country in which testing occurs affects the amount of second language (L2) interference experienced during first language (L1) processing.

Theories of bilingual memory differ with regard to how knowledge of L1 and L2 is stored in memory and how language knowledge is activated during processing. The RHM (Kroll and Stewart, 1994; Kroll et al., 2010; Poarch et al., 2015) proposes that bilinguals’ concepts (i.e., semantic representations) are shared between the two languages; however, lexical items in the two languages are stored separately, being connected by memory links. Memory links from L2 lexical items to L1 lexical items are claimed to be stronger than memory links from L1 lexical items to L2 lexical items. As bilinguals’ proficiency in L2 increases, the memory links between L2 lexical items and conceptual strengthen. Empirical support for the model continues to be observed [Sheng et al., 2013; Clenton, 2015; see also Pu and Tse (2014) for review]; however, critics of the RHM have argued that the model is unable to account for the patterns of bilingual language interference evidence that have been observed (Van Heuven et al., 1998; Spivey and Marian, 1999; Dijkstra et al., 2000; Marian et al., 2003, 2008; Duyck et al., 2007; Thierry and Wu, 2007; Qasem and Foote, 2010; Wang and Forster, 2010; Van Assche et al., 2012; Wang, 2013). For example, in experiments in which translation priming has been measured, significant priming effects are generally observed when bilinguals translate L1 to L2, but not when translating L2 to L1 (Wang and Forster, 2010; Wang, 2013). Wang (2013) concluded language dominance, rather than language proficiency, was the important factor in determine the size of the asymmetry in translation direction.

The BIA+ model of bilingual memory (Dijkstra and Van Heuven, 2002) claims that bilingual memory is organized with both languages being represented within the same distributed network composed of multiple levels (e.g., orthographic, phonological, semantic, etc.). During processing, memory representations for various language elements (e.g., phonemes, morphemes, words, etc.) from both languages become activated. The model also claims that the relative activation levels can be influenced by extra-linguistic factors, such as the task demands (Bijeljac-Babic et al., 1997; Van Heuven et al., 1998; Colomé, 2001; Dijkstra, 2001; Marian et al., 2003; Chee, 2006; Crinion et al., 2006; Kaushanskaya and Marian, 2007). Evidence for the model has come from experiments showing that the phonological attributes of one language can influence the processing of the word onset for words from the other language (e.g., Marian et al., 2003). A weakness of the BIA+ model is that it appears not to provide a satisfactory explanation of the facilitation effect in processing for words that are similar in form and meaning in a bilingual’s two language (i.e., cognates) as compared to typical translation equivalents, which have similar meaning but different phonological and orthographic form (i.e., non-cognates). Dijkstra and Van Heuven (2002) commented that the “available studies suggest that cognates have a special representation” (Dijkstra and Van Heuven, 2002, p. 185); however, it remains unclear whether different processing for cognates and non-cognates arises due to the shared phonological representations, the shared semantic representations, or both. Lemhöfer and Dijkstra (2004) suggested an account related to the fact that activation can flow both forward and backward in the network connected semantic and orthographic information. It is also unclear why the difference in processing for cognates versus non-cognates is reduced when sentence context is highly constraining (Libben and Titone, 2009).

A third, intriguing perspective on bilingual memory is one that predates both the RHM and the BIA+, but one that has received little attention in the empirical literature. It is the language mode theory proposed by Grosjean (1982, 2010), which claims that a bilingual does not constantly use one specific language processing mechanism, specifically that two languages of a bilingual can be activated as one system or activated separately from one another depending on the circumstance and the setting in which the language use is occurring. According to Grosjean (2013, p. 15), when a bilingual is preparing to speak, two operations occur. The first operation, called *language choice*, is selecting the language that the speaker is going to use. The language chosen is referred to as the *base language* (Grosjean, 2013, p. 15). This choice is usually determined by a number of factors such as the bilingual’s proficiency level in both languages, and the interlocutor (Grosjean, 2013). The second operation performed when speaking is deciding whether the other language is needed or not. This decision determines the bilingual’s language mode which Grosjean defines as “the state of activation of the bilingual’s languages and language processing mechanisms at a given point in time” (Grosjean, 2013, p. 15). Consequently, when making this decision, if the other language is not needed, its activation will be minimal, and the speaker will be in a monolingual mode. On the other hand, if the other language is needed, it will be activated, just less than the base language, and the speaker will be in bilingual language mode. Support for Grosjean’s (1982, 2010) language mode theory comes from research showing that bilinguals’ language processing can be influenced by the episodic context (Fishman, 1964, 1965; Giles et al., 1973; Sahgal, 1991; Siachitema, 1991; Grosjean, 2000; Reder et al., 2000; Duyck et al., 2008; Blanco-Elorrieta and Pylkkanen, 2015).

Grosjean’s (1982, 2010) language model theory suggests that proficiency levels of all the speakers in a setting can influence the activation levels of bilinguals’ languages in memory. For example, if a bilingual who is highly proficient in both languages is speaking to another bilingual who is also highly proficient in the same two languages, she may be more comfortable using both languages and have both languages highly activated in memory. However, if the recipient is not proficient in one of the two languages, the language that is not likely to be used in the setting may be lowered in activation in the bilingual speaker’s memory. There may also be individual differences across bilingual speakers in terms of how strongly they associate each of their languages with a particularly topic or life domains. For instance, young adolescents and young adults may use L1 in family situations (Siachitema, 1991) but use L2 in social events that include peers from other speech communities (Sahgal, 1991).

Of particular relevance to the present research, Grosjean (2013) also claimed that the location of a speaking event can also influence a bilingual’s language processing. He described two examples, an adult English–German speaker who was referred to as M. C. At the age of 26 years, M. C. was at a relatively low proficiency level in German because of his rare use of it in his daily life. However, when he moved to Germany at the age of 36 years, he started using the German language on a daily basis and became highly proficient in it. This change of routine in his language use caused a decline in his other two languages, English and French. Grosjean (2013) concludes that using a language in its native setting is quite different from using it in its non-native setting. Hence, location is assumed to have a noticeable effect on the language dominance of a bilingual as well as how active the two languages may be at a certain point of time.

Over the two decades, significant advances have been made in understanding the specific brain regions involved in the control of language interference for bilinguals (Green, 1986, 1998, 2011; Crinion et al., 2006; Luk et al., 2012; Green and Abutalebi, 2013; Abutalebi and Green, 2016). Green (1986, 1998, 2011) proposed that the regions of the brain evolved for general action control (i.e., cerebellum and subcortical areas, see also Green and Abutalebi, 2013; Abutalebi and Green, 2016). Recent brain imaging studies with bilinguals provide strong support for the view, confirming that language control is among the functions of the anterior cingulate cortex, the left prefrontal cortex, as well as the left and right inferior prefrontal areas (Crinion et al., 2006; Luk et al., 2012).

The purpose of the present research was to investigate the possibility that the amount of L2 interference that bilinguals experience when using their L1 depends on the context in which they are using their L1. Our primary research question was whether L2 interference that bilinguals experience would depend on the country in which they were tested, specifically when the countries differ in the frequency with which L2 is used. We examined processing by Arabic–English bilinguals, a group that has been included in relatively few prior studies (Dalrymple-Alford, 1968; Saegert et al., 1973; Liepmann and Saegert, 1974; Qasem and Foote, 2010; Coderre and Van Heuven, 2014; Blanco-Elorrieta and Pylkkanen, 2015; Boukadi et al., 2015). We reasoned that Arabic–English bilinguals provide an opportunity to test the prediction of Grosjean’s (1982, 2010) language mode theory, specifically the prediction that L2 would generally be activated more when the bilingual is in an environment in which L2 is frequently used versus an environment where L1 is used predominantly and L2 is rarely used.

In the present paper, we report the results of two experiments. In Experiment 1, we compared the processing of two groups of Arabic–English bilinguals a task similar to that used by Colomé (2001). One group was tested in Saudi Arabia in a setting where English was rarely used. The other group was tested in the United States in a setting where English was frequently used. In accordance with Grosjean’s (1982, 2010) language mode theory, we expected to observe less L2 interference during L1 processing in settings in which L2 is rarely used as compared with settings where L2 is the dominant language and is routinely used. In Experiment 2, we tested Arabic monolinguals in order to rule out the possibility that the pattern of processing differences in Experiment 1 were due to some factor(s) other than knowledge of English. In accordance with Grosjean’s (1982, 2010) language mode theory, we expected to observe more L2 interference for Arabic–English bilinguals tested in the United states than for those tested in Saudi Arabia. In contrast, in accordance with the RHM and the BIA+ model predicted, no effect of testing location was expected.

## Experiment 1

In Experiment 1, we compared processing for two groups of Arabic–English bilinguals in a task in which they used Arabic (L1) exclusively. One group was tested in Saudi Arabia and the other group was tested in the United States using the same portable computer. The task was modeled closely on that used by Colomé (2001). In the experiments reported Colomé (2001), participants viewed a series of pictures, each followed by a letter, and then were asked to judge whether the letter represented a phoneme that was contained in the Arabic word describing the picture. Our modification of the procedure was that following each picture, we presented a phoneme over headphones. This choice was motivated by the fact that Arabic and English utilize different writing scripts. In addition, we reasoned that auditory presentation of the phoneme may result in a more phonological salient stimulus as compared to the visual presentation of a letter in Colomé (2001). As in Colomé’s (2001) experiments, we compared three conditions that varied in terms of relatedness of the phoneme in relation to the picture name. In our conditions, the phoneme was either (a) contained in the L1 (Arabic) picture name, (b) contained in the L2 (English) picture name, and (c) unrelated to either the L1 or L2 picture names. For example, following the presentation of a picture of a basket, the participant would hear one of three phonemes: (a) /s/, which is contained in the Arabic word 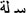 /b/ which is contained in the English word *basket*; and (b) /m/, which is not contained in either the Arabic word or the English word *basket*. We expected to replicate Colomé’s (2001) pattern of L2 interference, specifically greater processing difficulty when the phoneme was related to the L2 (English) picture name versus when it was unrelated to either the L1 or L2 picture name. In accordance with Grosjean’s (1982, 2010) language mode theory, we expected to observe more L2 interference for bilinguals tested in the United States than for those tested in Saudi Arabia.

### Method

#### Participants

A total of 105 Arabic–English bilinguals participated in the experiment. Fifty-five participants were living in Saudi Arabia and tested in that location. Sixty participants were living in the United States and tested there. All participants had normal or corrected-to-normal vision and reported no problems with their hands or fingers. **Table 1** displays descriptive statistics for the demographic variables for the two groups of participants. Participants received no compensation in exchange for their participation.

**Table 1:** Descriptive statistics for demographic variables for Arabic–Bilinguals in Experiment 1 by testing location.

	Tested in Saudi Arabia		Tested in the United States	
Characteristics	Mean	*SE*	Mean	*SE*
Age in years	30.74	1.02	28.08	1.15
Age of English acquisition	13.53	0.92	11.31	0.75
English proficiency	7.83	15	7.72	0.16
Hours of English weekly	43.46	1.78	54.06	4.33

*English proficiency was rated on a scale with *1* = *not at all proficient* to *10* = *extremely proficient**.

#### Materials

We initially identified phonemes that are shared by Arabic and English. These phonemes were /b/, /dʒ/, /f/, /h/, /j/, /k/, /l/, /m/, /n/, /s/, /ʃ/, /ð/, /w/, and /z/. We then identified concrete Arabic nouns containing one of these phonemes. From this initial working list, we eliminated those whose English translations were phonological similar to the Arabic translation equivalent and those that were more than one syllable. We also eliminated those items that could not be represented unambiguously in a line-drawing. Following these careful eliminations, we selected white-on-black line drawings for each of the words from a search on Google Images. We restricted our search to those drawings for which the copyrights permitted reuse for either commercial or non-commercial purposes. Of these, 6 were for a practice session, 27 were used for experimental trials, and 27 for filler trials, which were needed to balance the number of “yes” and “no” responses across the entire experiment. The images that were used for the experimental trials depicted one of the following: backpack, banana, basket, bucket, butterfly, candle, carrot, city, curtains, factory, farmer, fingerprint, flower, giraffe, ladder, library, mountains, mustache, newspaper, screwdriver, singer, skeleton, soldier, strawberry, wallet, woodpecker, and zipper. Some of the objects selected for the filler trials were one-syllable long. The images that were used for the filler trials depicted one of the following: balloons, broom, car, cell-phone, children, deer, dolphin, fish, gate, gun, jalapenos, lightbulb, ostrich, owl, pear, pineapple, scale, snake, square, table, telephone, tiger, toothbrush, tree, turtle, wheelchair, and zucchini. The images that used six practice trials depicted one of the following: bag, dress, fan, rabbit, star, and truck. Phonemes were recorded by a native speaker of Arabic who was highly proficient in English. The materials (i.e., recorded phonemes, images, and image-Arabic name pairings) were reviewed by four native speakers of Arabic and two native speakers of English who did not participate in any of the experiments to ensure that the phonemes were pronounced neutrally such that they would be perceived as representing an Arabic or an English phoneme. Arabic native speakers also reviewed the pictures and Arabic picture names to ensure that the picture name was strongly related to the picture. The English native speakers reviewed the pictures and English picture names to ensure that the picture names were strongly related to the picture. Estimates of English word frequency were obtained from the Corpus of Contemporary American English (COCA) (Davies, 2008/2017). COCA includes texts containing over 520 million words.

We collected demographic information from all participants. Participants were asked (in Arabic) to report their age, the age at which they began learning English, their weekly usage of English in hours, and their proficiency level in English on a 10-point scale (*1 = not at all proficient* to *10 = extremely proficient*). As shown in **Table 1**, the two groups did not differ significantly in age *t*(97) = 1.73, *p* = 0.09; sex (i.e., number of men and women), *t*(97) = 0.69, *p* = 0.49; L2 proficiency level *t*(97) = 0.48, *p* = 0.63; and age of L2 acquisition *t*(97) = 1.90, *p* = 0.06. As expected, we found that participants who were tested in the United States reported using English significantly more each week than those tested in Saudi Arabia *t*(97) = -2.28, *p* = 0.02.

#### Design

We used the same experimental design as Colomé (2001), which involved a 2 × 3 mixed factorial design with testing location as the between-subjects factor, having two levels (i.e., Arabic–English bilinguals tested in Saudi Arabia vs. Arabic–English bilinguals tested in the United States) and type of phoneme as the within-subject factor, with three levels (i.e., phoneme contained in the Arabic noun describing the picture for which a “yes” response was correct vs. phoneme contained in the English noun describing the picture for which a “no” responses was correct vs. phoneme contained in neither the Arabic nor English noun describing the picture for which a *no* response was correct). Each of the experimental and filler pictures was viewed three times, paired with a different phoneme. Experimental pictures were paired with three phonemes representing three conditions: (a) phoneme that occurred in the Arabic noun describing the picture; (b) phoneme that occurred in the English noun describing the picture; and (c) a phoneme that was not present in either the Arabic or English describing the picture. Because experimental trials involved two “no” conditions for every “yes” condition, filler trials involved two “yes” conditions for every “no” conditions (as was done in Colomé, 2001). For filler trials on which “yes” was the correct answer, the phoneme that was paired with the image was selected to occur in a variety of locations within the word (i.e., early, middle, and end of the word). The trials were presented in three blocks, ensuring that each experimental and filler picture was viewed once before any was viewed a second time, and each was viewed twice before any was viewed a third time.

#### Procedure

The research was reviewed and approved by the IRB at Oklahoma State University prior to participant recruitment. We recruited participants using a snowball technique. Steps were also taken to ensure that the experimental materials were appropriate. For all participants in both testing locations, the experimenter was a native speaker of Arabic who recruited and tested the participant using only Arabic. All conversation, information, feedback, and task material occurred in Arabic. Each participant was tested individually in an isolated quiet area in a single session lasting between 20 and 30 min. In each session, participants first reviewed booklet containing the black-on-white pictures paired with their Arabic, which was intended to familiarize participants with the nouns and their meanings. Second, they began the experimental task, read the instructions, had any questions answered, and completed a practice session composed of six items. Third, after any remaining questions were answered, participants performed the task, which was broken down into three blocks of 54 trials (i.e., 27 experimental trials and 27 filler trials) for a total of 162 trials. Each participant received a unique random order of trials in each block. We used E-Prime 2.0 Professional (Schneider et al., 2002) to control the presentation of stimuli and the recording of responses. Participants made responses using the computer keyboard. Each trial involved the presentation of the picture for 300 ms followed by a blank screen 100 ms followed by the auditory presentation of the phoneme (whose duration ranged from 0.20 to 0.23). Participants were permitted 3 s to respond. After each response, participants received feedback on the computer screen, which indicated whether their response had been correct, incorrect, or timed out written in Arabic. The number of trials for which *yes* and *no* were correct answers was matched exactly overall and closely matched for each of the 14 phonemes used in the experimental trials. The last part of each sessions involved participants completing the demographic questionnaire in Arabic.

#### Data Analysis

Error rate and reaction time data were analyzed using analyses of variances (ANOVAs) in which both subjects and pictures were treated as random effects in accordance with recommendations by Clark (1973). Statistical analyses were conducted using SPSS (21.0).

### Results and Discussion

Participants’ response times and accuracy were examined for outliers. We followed Colomé’s (2001) procedure. First, we identified pictures for which there were high error rates across participants. Two pictures had overall error rates above 30% (i.e., woodpecker and ladder). We excluded these trials from the dataset. Second, using the trimmed dataset, we examined error rates for each participant and excluded participants with error rates higher than 30%. Data from 16 participants were excluded, so that 50 participants remained in the group tested in Saudi Arabic and 49 tested in the United States. Lastly, using the trimmed dataset with 99 participants, we excluded response times shorter than 200 ms and longer than 3000 ms. The mean error rates and response times were calculated for each participant by condition. These results are displayed in **Table 2**. The pattern of error rates made by both groups of participants indicated the presence of L2 interference during processing in the task. The main effect of phoneme type was significant, *F*_1_(2,194) = 5.04, *p* = 0.007, η^2^ = 0.24, *F*_2_(2,96) = 4.70, *p* = 0.01, η^2^ = 0.16. This main effect stemmed from significantly more errors occurring in the English phoneme condition than the unrelated phoneme condition, *F*_1_(1,75) = 18.77, *p* < 0.001, η^2^ = 0.20, *F*_2_(1,49) = 6.83, *p* = 0.012, η^2^ = 0.12 and significantly more errors occurring in the Arabic phoneme condition than in the unrelated phoneme conditions, *F*_1_(1,75) = 13.95, *p* < 0.001, η^2^ = 0.16, *F*_2_(1,49) = 7.28, *p* = 0.01, η^2^ = 0.13. Errors in the Arabic phoneme condition did not differ significantly from those made in the English phoneme conditions, *F*s < 1. The main effect of subject type and the interaction between subject type and phoneme type were not significant, *F*s < 1 and *F*_1_(1,97) = 3.12, *p* = 0.08, *F*_2_(1,48) = 1.40, *p* = 0.24, respectively.

**Table 2:** Mean error rates in percent and response times in milliseconds for Arabic–English bilinguals tested in Saudi Arabia (*n* = 50) and the United States (*n* = 40) by condition in Experiment 1.

	Saudi Arabia				United States			
Phoneme type	Mean error	*SE*	Mean RT	*SE*	Mean error	*SE*	Mean RT	*SE*
Arabic phoneme	12.88	1.33	680.89	28.53	15.76	1.62	691.44	28.82
English phoneme	12.56	1.19	950.64	39.81	16.33	1.49	954.87	40.21
Unrelated phoneme	10.40	1.40	936.81	41.47	11.92	1.62	960.80	41.90

*RT = response time in milliseconds*.

The pattern of results for response times did not indicate presence of L2 interference during the experiment. Response times were significantly faster in the Arabic phoneme condition than in either the English phoneme conditions, *F*_1_(1,75) = 232.40, *p* < 0.001, η^2^ = 0.77, *F*_2_(1,49) = 177.56, *p <* 0.001, η^2^ = 0.78 or the unrelated phoneme condition, *F*_1_(1,75) = 200.99, *p* < 0.001, η^2^ = 0.73, *F*_2_(1,49) = 221.78, *p <* 0.001, η^2^ = 0.82. Response times in the English phoneme condition did not differ significantly from those in the unrelated phoneme condition, *F*s < 1. The main effect of phoneme type was significant, *F*_1_(2,194) = 209.64, *p* < 0.001, η^2^ = 0.70, *F*_2_(2,96) = 122.00, *p <* 0.001, η^2^ = 0.85. The main effect of subject type and the interaction between subject type and phoneme type were not significant, *F*s < 1.

#### Correlational Analyses

We explored the extent to which both groups of participants’ English age of acquisition, proficiency, and hours of weekly usage were related to their performance in the English phoneme condition in the experiment, specifically their error rate and response times. These analyses yielded significant results for bilinguals tested in the United States but not for bilinguals tested in Saudi Arabia. For those participants living in the United States, error rates in the English phoneme condition was higher when for participants who reporter greater use of English each week, *r* = 0.38, *p* = 0.007. Correlational analyses between English age of acquisition, proficiency, and usage and performance in Arabic phoneme and unrelated phoneme conditions were not significant.

We also explored the extent to which the printed frequency of the English picture name, which never occurred in the experiment, but may have been activated in bilinguals’ memory, was related to both groups of participants’ performance in the English phoneme condition. The analyses yielded significant results for both groups of participants. Response times in the English phoneme conditions were significantly slower for trials with pictures whose English names were higher in frequency. The correlations were significant for participants tested in the United States, *r* = -0.47, *p* = 0.05, for participants tested in Saudi Arabic, *r* = -0.40, *p* = 0.02 and overall, *r* = -0.43, *p* = 0.002. Correlational analyses frequency of the English picture name and response time in the Arabic phoneme and unrelated phoneme conditions were not significant. There were no significant results for analyses involving error rates.

In sum, we found evidence for L2 interference in mean error rates, but not in mean response times. However, in correlational analyses of response time, we found additional indications of activation of L2 during the task. Participants’ error rates were higher in the English phoneme condition when their weekly usage of English was higher, and participants’ response times in the English phoneme condition were slower when the English picture name was higher in frequency. The purpose of Experiment 2 was to rule out the possibility that the results obtained in this experiment were the result of aspects of the methodological procedure or materials, rather than the result of the participants’ Arabic–English bilingual status.

## Experiment 2

We aimed to confirm that our results with bilingual participants in Experiment 1 occurred because of their L2 (English) knowledge. We employed a strategy similar to that used by Colomé (2001). We implemented the experiment again with individuals with little or no knowledge of English whose L1 was Arabic. We expected to observe no processing differences for the English phoneme and unrelated phoneme conditions either in error rates or response times.

### Method

#### Participants

Twenty-four native speakers of Arabic (10 men, 14 women) for whom Arabic was the only language known fluently participated in the experiment for no compensation. The mean age of participants was 41.42 years (*SE* = 1.85). All participants had normal or corrected-to-normal vision, and none suffered from problems moving their hands or fingers.

#### Design, Procedure, and Data Analysis

The design, procedure, and data analysis were the same as in Experiment 1.

### Results and Discussion

Data were trimmed and analyzed in the same manner as in Experiment 1. **Table 3** displays mean reaction times and error rates by condition. The results indicated a pattern of results that differed from Experiment 1. ANOVAs for error rates showed that the main effect of phoneme type was not significant, *F*_1_(2,48) = 1.77, *p* = 0.18, *F*_2_ < 1^1^. ANOVAs for response times showed the main effect of phoneme type differed significantly for the three conditions, *F*_1_(2,46) = 58.00, *p* < 0.001, η^2^ = 0.06, *F*_2_(2,48) = 50.58, *p <* 0.001, η^2^ = 0.68. Responses in the *yes* condition were significantly faster than responses in each of the *no* conditions: Arabic phoneme conditions versus unrelated phoneme condition: *F*_1_(1,23) = 63.63, *p* < 0.001, η^2^ = 0.73, *F*_2_(1,24) = 66.18, *p* < 0.001, η^2^ = 0.73 and Arabic phoneme conditions vs. English phoneme condition: *F*_1_(1,23) = 67.63, *p* < 0.001, η^2^ = 0.73, *F*_2_(1,24) = 68.54, *p* < 0.001, η^2^ = 0.74.

**Table 3:** Mean error rates in percent and response times in milliseconds for Arabic monolinguals by condition in Experiment 2.

	Errors in percent		Response time in milliseconds	
Phoneme type	Mean	*SE*	Mean	*SE*
Arabic phoneme	16.50	1.95	718.49	42.28
English phoneme	20.17	1.83	1056.82	68.92
Unrelated phoneme	15.83	2.15	1041.75	68.50

## General Discussion

The research investigated whether the testing location influenced the amount of L2 (i.e., English) interference experienced by Arabic–English bilinguals during a processing task involving only L1 (Arabic). In Experiment 1, the processing for two groups of bilinguals who were closely matched on demographics and English-related variables was compared. One group of bilinguals was tested in Saudi Arabia and the other in the United States. The results showed that testing location did not influence processing. Both groups of bilinguals experienced L2 interference in error rates, as they were more likely respond *yes* rather than *no* in the English phoneme condition. Although mean response times were not significantly influenced by L2 interference, the results of correlational analysis provided evidence for some activation of English during processing. Response times in the English phoneme condition were slower (i.e., indicating more L2 interference) for individuals who reported using English more each week. Also, response times were slower in the English phoneme condition on trials on which the English picture name was higher in frequency. In Experiment 2, we showed a group of monolingual participants carrying out the same task showed no significant processing differences between the English and unrelated phoneme conditions and showed no significant correlations involving the English phoneme condition. These results can be viewed as inconsistent with Grosjean’s (1982, 2010) language mode theory, which emphasized the role of setting in determining not only the behavior of bilinguals, but also the extent to which a bilingual’s two languages are activated in memory. On the other hand, Grosjean (1982, 2010) suggested that L2 is never completely deactivated; thus, the view can account for the observed L2 interference in Experiment 1.

The results are compatible with the RHM and BIA+ model, which did not predict an effect of testing location on L2 interference. The L2 interference observed in the present result can be viewed as unexpected from the perspective of the RHM, which predicts that L2 interference might occur during L1 processing, particularly for highly proficient bilinguals, as the participants in our Experiment 1 were. Because the RHM makes the specific claim that memory links between L1 and L2 are particularly weak, the fact that L2 interference was observed for both groups of bilinguals was unexpected because processing by bilinguals in a task exclusively in L1 would be expected to active L2 words relatively weakly.

The present results appear most compatible with the BIA+ model, which predicts that there would be greater L2 interference during L1 processing when there is greater interrelatedness in a bilinguals’ two languages, because “the larger the overlap between the input string and a representation in the mental lexicon, the more the internal representation is activated” (Dijkstra and Van Heuven, 2002, p. 182). A comparison of the present results with those of Colomé (2001) indicates the amount of L2 interference observed in the studies may have differed. Colomé (2001) found effects in mean response times in two experiments and in mean error rates in one experiment, the present study observed L2 interference in mean error rates. Overall, the error rates in Colomé’s (2001) experiments with Catalan–Spanish bilinguals were approximately 10% lower than in the present experiment. Catalan and Spanish five vowels out of a total of eight and share most, if not all, consonants, and have approximately 85% overlap in the similarity of lexical items (Simons and Fennig, 2017). They also share the same orthography. Arabic and English share far fewer phonemes and lexical items. Dijkstra and Van Heuven (2002) pointed out that if “the two languages differ with respect to their input codes (e.g., letter sets), the activated set of neighbors may become much smaller” (p. 183). It is possible that the difference between the results of the present results those of Colomé’s (2001) were due only to the differences in methodology (i.e., auditory presentation of phoneme instead of visual presentation of letter representing a phoneme due to the fact that Arabic and English use different writing scripts).

The possibility that the amount of L2 interference may depend on the amount of overlap between a bilingual’s two languages is intriguing and merits further study. Arabic–English bilinguals are also likely different from Catalan–Spanish bilinguals in how the two languages are used in daily life. Sebba (2011) has described Catalan–Spanish bilinguals are *societal bilinguals*, as at least 97% of the population of Catalonia comprehends both Catalan and Spanish, and 85% of the population speaks the two languages (Simons and Fennig, 2017). In contrast, Arabic–English bilinguals have been referred to as *individual bilinguals* (Hoffmann, 2014), as they belong to a community which primarily uses Arabic as its main language and English is acquired to be used in specific domains (e.g., school, work, etc.). The processing implication is that there may be less L2 activation during L1 processing for individual bilinguals (i.e., Arabic–English bilinguals) than for societal bilinguals (i.e., Catalan–Spanish bilinguals).

The present research has at least three limitations. Experiment 1 compared different participants tested in the different locations. It is possible that differences between the groups unrelated to the experiment contributed to the failure to observe a difference related to testing location. A stronger test of the effect of location on L2 interference would involve testing the same group of participants in the two locations. Although it was not possible for us to carry out a completely within-subjects design for the present research, we look forward to future research in which such a design has been implemented. In this research, the challenge will be to prevent participants from discovering that the study examines L2 activation, as awareness of the purpose of the study may influence performance. A second limitation is that we relied only on self-report measures of L2 proficiency. More sensitive measures of L2 proficiency may have been able to reveal a more fine-grained relationship between proficiency and performance in the processing task. Further, it is possible that self-report measures of L2 proficiency may differ for the two types of bilinguals in Experiment 1. A third limitation is our use of non-standardized images for stimuli in the experiment, a procedure also employed by Colomé (2001). In future research, the use of standardized images may lead to less variance in performance across participants.

The extent to which differences in the results of the present research and those of Colomé (2001) reflect differences in the interrelatedness of the bilinguals’ two languages is worthy of future research. Research is needed to determine what factors determine how much L2 interference occurs during bilingual language processing (e.g., Gerard and Scarborough, 1989; Marian et al., 2003; Qasem and Foote, 2010; Coderre and Van Heuven, 2014). In light of the significant correlations in the present research between response times and English picture name word frequency, it is possible that future research will find that the amount of L2 interference depends directly on item-specific characteristics as well as the interrelatedness between words in the bilinguals’ two languages with regard to the specific characteristics being examined, which would directly relate to the numbers of types of lexical representations activated following the processing of a L1 word.

In sum, the present research investigated whether testing location influenced the amount of L2 interference experienced by Arabic–English bilinguals in a task in which only L1 was used. Unlike prior research with Catalan–Spanish bilinguals by Colomé (2001) who observed L2 interference in mean error rates and mean response times, the present research observed L2 interference in mean error rates and in correlations between individual participants’ response times and their weekly usage of L2, showing more L2 interference when more L2 was used each week. We also observed correlations between response times for individual items and the frequency of the L2 picture name, showing more L2 interference when the L2 picture name was higher in frequency. We believe that the results are best explained by the BIA+ model, as it claims that the amount of L2 activation that occurs is a direct result of the interrelatedness of the words in the bilingual’s two languages. We hope the research provides an impetus for (a) future research investigating the possibility that the amount of L2 interference occurring during L1 processing depends on the interrelatedness of a bilingual’s two languages and (b) future research in understudied languages, such as Arabic.

## Ethics Statement

This study was carried out in accordance with the recommendations of the Human Research Participant guidelines of the American Psychological Association with written informed consent from all subjects. All subjects gave written informed consent in accordance with the Declaration of Helsinki. The protocol was approved by the IRB at Oklahoma State University.

## Author Contributions

TA conducted the research as part of her fulfillment of the Ph.D. in English. She developed the hypotheses, collaborated on the design, collected and analyzed the data, and wrote the first draft of the manuscript. SK supervised the dissertation and collaborated on the design, programmed the software, assisted in the analysis of the data, and revised the manuscript.

## Conflict of Interest Statement

The authors declare that the research was conducted in the absence of any commercial or financial relationships that could be construed as a potential conflict of interest.

## Footnotes

1 An alternative approach to the data analysis included conducting *a priori* comparisons. Pairwise comparisons of error rates yielded no significant results: Arabic phoneme conditions vs. the unrelated condition, *F*s < 1, Arabic phoneme condition vs. English phoneme condition, *F*_1_(1,23) = 3.65, *p* = 0.08, *F*_2_(1,24) = 1.51, *p* = 0.23, and English phoneme condition vs. the unrelated condition, *F*_1_(1,23) = 3.90 *p* > 0.06, *F*_2_(1,24) = 2.16, *p* = 0.16.
